# Progress in Medicine

**Published:** 1897-04-17

**Authors:** 


### Progress in Medicine.

### RENAL MEDICINE.

*Treatment.*—The use of saline injections in uræmia has not been universally successful, but some recoveries in thoroughly hopeless cases have been recorded. Richardière[1] finds that among symptoms following these injections are the following favourable ones. The temperature rises a degree or two, the pulse rate is diminished, the respirations become regular, diuresis and a remedial diarrhœa are increased. Moreover, the injections of artificial serum are perfectly harmless, and even when thrown into œdematous tissue they cause no local inflammation if made with antiseptic precautions, though there may be some little pain produced. He narrates two cases with favourable results. The first patient was comatose with Cheyne Stokes breathing and rales all over the chest. He was bled ten ounces, and twenty-six ounces of Hayem's serum was injected into the cellular tissue. Great improvement followed, but a relapse occurred some days later, and the process was repeated with two injections of the same amount, after which the uræmia disappeared permanently. Lochelongue[2] gives the history of the use of this treatment. In fevers there is first a rigor and rise of temperature, which is quickly followed by a fall to normal and general improvement. He advises venesection before the injections in uræmia, and thinks that subcutaneous injections should always be used instead of intravenous ones, if it is possible to wait an hour or two, as the latter are not without dangers. L. Guthrie,[3] in discussing the treatment of interstitial nephritis in children, takes as his chief rule, "watch the heart and pulse," so as to use heart tonics and vascular constrictors in the earliest stages for the promotion of diuresis, and again in the last stage when compensation has given way. In the period of excessive cardiovascular hypertrophy, when there is danger from excessive tension, vasodilators and agents which act on the skin and bowels are indicated. The cause of these rare cases, is, he thinks, neither scarlatina, uric acid, or a previously existing white kidney, but usually an acute nephritis, produced by cold or toxins. Emile Julia[4] speaks highly of the application of pilocarpine ointment, about a grain to the ounce, in various forms of nephritis. It was rubbed into the dorso-lumbar region daily and a covering of waxed linen applied afterwards. The author claims that œdema, dyspnœa, and a large amount of albumen disappear under this method.

Tubercular disease of the kidney, when primary, may be removed in many cases by medical treatment. If this fails the question of surgical interference must be considered. Tuffier[5] even then only advises an operation if the general health is failing from hæmaturia, pain, infection, or intoxication. Thus hæmaturia may be so copious and persistent as to endanger life, or pain may produce the same risk. More often the retention of septic products produces a hectic state, which may justify the excision or drainage of the diseased organ. The syphilitic kidney, as distinct from that due to the mercury employed, may occur at any stage of the exciting disease. An article in the *Therapeutic Gazette* (February 17th), describes the symptoms as including frequent micturition, œdema, headache, and dyspepsia, with albumen, blood, and various kinds of casts. Pathologically, the changes are those of a form of acute nephritis, but in late stages we may find amyloid or interstitial changes as well as gummata. Welander, on the other hand, thinks these attacks are rare, and that mercury itself causes frequent casts and albumen; but the prognosis is good, as it is, indeed, in real syphilitic nephritis, though mercury should be given with caution when this development has occured, more especially as toxic effects arise when the kidneys are crippled. Most observers, however, agree that true syphilitic nephritis is cured by ordinary mercurial treatment Probably the best line is to continue treatment steadily with the smallest possible dose of the drug, such as by the mercury and chalk pill, or, in the later stages, by iodide of potash. The hæmaturia, seen in another infective disorder, malaria, also requires specific treatment, such as quinine and arsenic, for its permanent cure, though for temporarily stopping attacks, Guice,[6] and others, insist on the great value of oil of turpentine. This is given to the extent of ten drops, every three or four hours, together with sulphate of magnesia.

**Urology.**—Bard[7] endeavours to estimate the indications given by the urine in very chronic cases of parenchymatous nephritis. There are instances where large amounts of albumen are passed for long periods, though the patient enjoys excellent health, and in these he thinks we have not atrophied tubules surrounded by fibrous tissue, but regenerated imperfect epithelial cells, like those produced over superficial burns of the skin. He regards the casts as of prime importance in the prognosis of these cases, and where after repeated examinations granular and colloid casts are absent, though albumen persists, he considers that the progress of the disease has ceased, and the cicatricial condition has been produced. The granular casts, which show a very active form of disease, are the most opaque and solid. However, other observers, by the aid of the centrifuge, continue to discover various casts, even in the urine of normal individuals.

Thus G. Barrie[8] found in fifty healthy persons thirteen cases where hyaline casts were present, small granular ones in six, and epithelial in one. Twenty out of the fifty showed some trace of albumen. In another group with none of the general symptoms of renal disease, but suffering from other complaints, he discovered casts in fifteen per cent. of all ages. Shattack and L. Carter Gray have also met with them in many persons who could not be supposed to have Bright's disease. On the other hand, Barrie, Senator, and Johnson describe cases where both casts and other symptoms of Bright's disease have been present, but complete and absolute recovery has taken place afterwards. Still it must be remembered that casts and albumen may be absent, though advanced disease exists, as shown by deficient excretion of solids, and by constitutional symptoms. On the whole it is clear that in forming a prognosis, the presence of casts or of albumen is only one of many factors, which should be taken into careful consideration. Even apart from the question of physiological albumen there are many possibilities of error, such as the presence of symptomatic albumen from heart or lung disease, and in women from leucorrhœa; or the error from estimating mucin, pepton, or albumose as true albumen. Arnozan argued at the French Congress that all inflamed epithelia exude albumen, and he regards albumen in some forms of acute nephritis as a mere inflamatory product, while in kidney lesions, which are clearly degenerative rather than inflammatory, such as phosphorus poisoning or cancer, there is little or no albumen. If the renal filter is diseased, it is said to let albumen slip through, but this is just the time when it fails to pass the soluble substances, such as urea or chlorides. He adds, that as a matter of fact albumen is produced by so many causes that it is impossible to attach to it any prognostic value of its own apart from the other circumstances of the case as aids to prognosis. Talamon, in a paper read at the same time, suggests that if, on the addition of nitric acid to albuminous urine, a layer of indican forms beneath the precipitate, and the latter contracts into a large clot, the prognosis is bad, while if a layer of uric acid forms on the top of the albumen, the attack is mild. A decrease, too, of the serin, as opposed to the globulin is another bad sign; so, too, a large amount of albumen where the solids of the urine are deficient, is unfavourable. Still a small amount of urea does not of itself prove aggravation of the disease, for it may depend on scanty nourishment, such as a milk diet. Heredity, or the age of the patient when past early adult life, may contribute unfavourable elements to a prognosis. Albumen in an early stage of gout may disappear, that in the latter periods, combined with heart symptoms, is serious, and albumen occurring with sugar, indicates a mild form of diabetes; if it comes on late in the disease, or replaces the sugar, the outlook is serious. In phthisis, besides the mild pretubercular form, we have the grave types, called the purulent, the persistent hæmorrhagic and polyuric. Teissier holds that some forms of albuminuria never develop into Bright's disease. To this class belong certain kinds of intermittent or cyclic type, especially the pregouty, seen in children of arthritic parents, and the pretubercular, which shows that the organism is impregnated with tuberculin. The prognosis as to future kidney disease is excellent, though the pretubercular is an indication of an even more serious affection. He gives the name of Pavy's disease to pregouty albuminuria, he denies that those subject to it tend to develop interstitial nephritis later on, and reports that of twenty-eight patients under observation none have died, and twenty-four have been free from albumen for several years. It is a cyclic or intermittent affection, and the cycle, or course of an attack, includes an increase of colouring matter, and urates with albumen and low tension pulse together a greater toxicity of the urine. He has watched many such patients for ten years, and regards them as suitable for insurance, if there is no family history of Bright's disease, and no heart changes.

Chvostek and Stromayer[9] believe that albumosuria may be used for the detection of gastric or tubercular ulcers of the intestine. For, if a meal of peptones or somatose be given to healthy persons, albumosuria does not appear. If it follows such a meal, they find that ulceration is present.

Kenneth Watson points out[10] that three sources have been ascribed to albumosuria, the blood, morbid conditions of the tissues, and the diseased state of the gastro-intestinal organs. Possibly any of these may produce it.

[1] Union Med., Dec. 5. [2] B. M. J., Jan. 30. [3] Lancet, March 13. [4] N. Y. Med. Jour., Dec. 26. [5] Med. Week. [6] Therap. Gazette, Feb. 15. [7] Med. Chron., Dec. [8] Med Rec., Jan. 23. [9] B. M. J., Feb. 13. [10] B. M. J. Jan. 16.

